# Initial Treatment and Outcomes of Complete Hydatidiform Mole in Women 40 Years or Older: A Multicenter Cohort Study

**DOI:** 10.3390/cancers17193125

**Published:** 2025-09-26

**Authors:** Cecília Canêdo Freitas Desmarais, Izildinha Maestá, Sue Yazaki Sun, Jorge de Rezende-Filho, Roberto Antonio de Araújo Costa, Lawrence Hsu Lin, Mariza Branco-Silva, Neil S. Horowitz, Kevin M. Elias, Antonio Braga, Ross S. Berkowitz

**Affiliations:** 1 Postgraduation Program in Tocogynecology, Medical School, São Paulo State University (UNESP), Av. Professor Mário Rubens Guimarães Montenegro, s/n°, Botucatu 18618-687, SP, Brazil; ceciliacanedo@gmail.com (C.C.F.D.); branco.silva@unesp.br (M.B.-S.); 2 Botucatu Trophoblastic Disease Center, Medical School, São Paulo State University (UNESP), Av. Professor Mário Rubens Guimarães Montenegro, s/n°, Botucatu 18618-687, SP, Brazil; 3 São Paulo Hospital Trophoblastic Disease Center, Paulista School of Medicine, São Paulo Federal University, R. Napoleão de Barros, 715-Vila Clementino, São Paulo 04024-002, SP, Brazil; sueysun@gmail.com; 4 Rio de Janeiro Trophoblastic Disease Center, Maternity School, Rio de Janeiro Federal University, Rua das Laranjeiras, 180, Laranjeiras, Rio de Janeiro 22240-003, RJ, Brazil; rezendef@me.ufrj.br (J.d.R.-F.); bragamed@yahoo.com.br (A.B.); 5 Medical School, São Paulo State University (UNESP), Av. Professor Mário Rubens Guimarães Montenegro, s/n°, Botucatu 18618-687, SP, Brazil; roberto.costa@unesp.br; 6 Department of Pathology, Massachusetts General Hospital, Harvard Medical School, 55 Fruit Street, Boston, MA 02114, USA; 7 New England Trophoblastic Disease Center, Division of Gynecologic Oncology, Department of Obstetrics, Gynecology and Reproductive Biology, Brigham and Women’s Hospital, Dana Farber Cancer Institute, Harvard Medical School, 450 Brookline Avenue, Boston, MA 02215, USA; nhorowitz@mgh.harvard.edu (N.S.H.); ross_berkowitz@dfci.harvard.edu (R.S.B.); 8 Section of Gynecologic Oncology, Obstetrics and Gynecology Institute, Taussig Cancer Institute, Lerner Research Institute, Cleveland Clinic Foundation, Cleveland, OH 44195, USA; keliasmd@outlook.com; 9 Department of Maternal and Child Health, Postgraduate Program in Medical Sciences, School of Medicine, Fluminense Federal University (UFF), Niterói 24070-090, RJ, Brazil; 10 Postgraduate Program in Applied Health Sciences, University of Vassouras, Vassouras 27700-000, RJ, Brazil

**Keywords:** complete hydatidiform mole, advanced maternal age, uterine evacuation, hysterectomy, gestational trophoblastic neoplasia

## Abstract

**Objectives**: To evaluate the potential associations of the type of complete hydatidiform mole (CHM) initial treatment (hysterectomy or uterine evacuation) with GTN development, need for chemotherapy, and treatment outcome in women aged ≥ 40 years. **Methods**: This multicentric retrospective cohort study included women ≥ 40 years with CHM, initially treated between 1990 and 2018, at four different centers. Data collected included patient demographics and clinical characteristics. The outcome variables were post-CHM GTN development, need for chemotherapy for hCG normalization, surgical complications, and time to remission. Univariate and multivariate analyses were performed using chi-square, Mann–Whitney, Fisher’s exact tests, and Poisson regression. **Results**: 275 women with CHM aged ≥ 40 years were included in the analysis. Median patient age was significantly higher among hysterectomy patients (47 × 44 years, *p* = 0.01). Multivariate analysis showed that compared with uterine evacuation (244/275, 89%), hysterectomy (31/275, 11%) was associated with an 83% lower risk of GTN [RR = 0.17 95% CI = (0.04–0.71); *p* = 0.015] and a 92% lower risk of requiring chemotherapy [RR: 0.08 (0.01–0.64), *p* = 0.016]. Median time to hCG normalization did not statistically differ between treatments. No significant differences were observed between hysterectomy and uterine evacuation in terms of FIGO staging (*p* = 0.221) or prognostic risk score (*p* = 0.576). Resistance to first-line chemotherapy (17/72; 23.6%) and relapse (3/72; 4.1%) were observed only in patients undergoing initial uterine evacuation. Hysterectomy complications occurred in 45.1% (14) of the patients. **Conclusions**: CHM initial treatment with hysterectomy was associated with a lower risk for GTN occurrence and need for chemotherapy in women aged 40 years or older. However, shared decision-making about surgery should be tailored to each patient and their risk factors and preferences. Further, larger controlled studies are required to support our findings.

## Simple Summary

Complete hydatidiform mole is a rare condition that can lead to gestational trophoblastic neoplasia (GTN), especially in women over 40. These women are at higher risk of complications and may require chemotherapy. This study investigated whether removing the uterus (hysterectomy) instead of standard uterine evacuation could reduce the chance of developing GTN and the need for chemotherapy. The study included women from different treatment centers and compared their outcomes based on the type of initial treatment they received. The findings showed that hysterectomy was associated with a lower risk of GTN and less need for chemotherapy, but it also carried a risk of surgical complications. These results suggest that hysterectomy may be a valid option for older women who do not wish to preserve fertility, reinforcing the importance of discussing treatment decisions based on personal health risks and preferences.

## 1. Introduction

Hydatidiform mole (HM) is caused by abnormal oocyte fertilization and is characterized by marked proliferation of the placental trophoblast and increased production of human chorionic gonadotropin (hCG). The types of hydatidiform mole, complete mole (CHM) and partial mole (PHM), are distinguishable by their clinical characteristics, chromosomal patterns, and histopathological features [1,2]. The overall incidence of CHM in the general population is estimated as 1 for every 1423 pregnancies [3].

Post-molar gestational trophoblastic neoplasia (GTN) develops in approximately 15–20% of women with a CHM and in 0.5–5% of those with a PHM, often necessitating chemotherapy [4,5].

Advanced maternal age is a well-established HM risk factor, with women ≥ 40 years facing nearly twice the risk of developing CHM compared to those aged 20–39 years [6]. Moreover, older women are at significantly increased risk of developing post-molar GTN. In women of advanced maternal age, the likelihood of GTN following CHM ranges from 30 to 50% [3,7].

In CHM women ≥ 40 years, hysterectomy, rather than uterine evacuation, has been recommended as an option for the initial treatment of patients no longer wishing to conceive [8,9], given the potential benefit of removing the affected uterus to prevent local invasion and subsequent malignant transformation. However, whether hysterectomy effectively prevents post-molar GTN remains debatable [7,10,11].

Few studies have specifically targeted patients at least 40 years old, and most included patients with invasive mole (IM) in the hysterectomy group, which could increase the probability of post-molar GTN and potentially introduce bias. We should keep in mind that an IM is an HM that penetrates the myometrium, with potential for local invasion, already representing a form of GTN and often requiring chemotherapy. Furthermore, previous studies have primarily focused on patients from developed regions, and most were conducted in a single center [3,6,7,11], restricting the generalizability of findings. A multicenter study involving diverse regions and contexts may advance the understanding of CHM progression and treatment in women ≥ 40 years.

Within this framework, the purpose of this study was to evaluate the potential associations of the type of initial treatment for CHM (hysterectomy or uterine evacuation) with the need for chemotherapy, GTN development, and treatment outcomes in this population. Additionally, surgical complications associated with hysterectomy were assessed.

## 2. Methods

### 2.1. Study Design and Setting

This multicentric retrospective cohort study included women aged ≥ 40 years with CHM receiving initial treatment between 1990 and 2018 at the following centers: New England Trophoblastic Disease Center (Harvard Medical School, USA); Botucatu Trophoblastic Disease Center (Botucatu Medical School, São Paulo State University/UNESP); Rio de Janeiro Trophoblastic Disease Center (Federal University of Rio de Janeiro/UFRJ, Fluminense Federal/UFF University); and São Paulo Hospital Trophoblastic Disease Center (Paulista School of Medicine, Federal University of São Paulo/UNIFESP).

### 2.2. Inclusion and Exclusion Criteria

All four study centers specialize in gestational trophoblastic diseases and use the same diagnostic criteria and treatment strategies to manage CHM. The hCG reference value used in all chemiluminescent immunoassays was <5.0 IU/L.

In all cases, CHM diagnosis was established based on pre-evacuation quantitative hCG testing and ultrasound signs suggestive of CHM, and post-evacuation histopathological confirmation [1,12]. p57 immunohistochemistry (KIP2) [13] was used only when morphologic examination was inconclusive.

Exclusion criteria were prophylactic chemotherapy at the time of molar evacuation, loss to postmolar follow-up, and IM at hysterectomy for CHM.

All participants underwent initial assessment, initial treatment (hysterectomy or uterine evacuation), subsequent treatment and follow-up at one of the participating centers. Hysterectomy was offered as an option for the initial management of CHM, with the decision made jointly with the patient, considering individual risk factors and personal preferences.

GTN was defined according to the FIGO/WHO criteria as persistent hCG plateau over ≥3 weeks, hCG re-elevation over ≥2 weeks, or histologic diagnosis of choriocarcinoma [9,14]. Following GTN diagnosis, treatment options—hysterectomy or chemotherapy—were discussed, and decisions were once again shared with patients, considering clinical risk factors and individual preferences.

### 2.3. Data Collection and Study Variables

Medical records, both in paper and electronic form, were reviewed for data on maternal age, gravidity, parity, ultrasound diagnosis, gestational age at CHM diagnosis (calculated based on the last menstrual period), uterine size, presence of vaginal bleeding/spotting, theca-lutein ovarian cysts > 6 cm, and pre-evacuation hCG levels. Medical complications at presentation evaluated included hemorrhage leading to hemodynamic instability, hyperemesis (nausea and vomiting ≥ 5 times/day requiring antiemetics) [15], preeclampsia (blood pressure ≥ 140/90 mmHg with proteinuria or target organ damage), overt hyperthyroidism (low or undetectable TSH levels and elevated free T4 levels, with or without classic symptoms) [16]; and acute respiratory distress syndrome (ARDS). Data on associated gynecological disorders (e.g., uterine fibroids, adenomyosis, cervical intraepithelial neoplasia) were collected only from patients undergoing hysterectomy.

### 2.4. Outcomes

The outcome variables were GTN development, need for chemotherapy (increased hCG levels over two consecutive weeks or hCG plateauing for three weeks after CHM initial treatment), and time to remission. Hysterectomy complications were categorized according to the Clavien–Dindo system (2004) [17].

### 2.5. Follow-Up

Follow-up duration was at least 6 or 12 months after hCG normalization, depending on whether normalization occurred after CHM spontaneous remission or GTN, respectively.

### 2.6. Statistical Analysis

Statistical analyses were performed considering the type of initial treatment (hysterectomy or uterine evacuation) as the main independent variable. Potential confounding variables previously described in the literature [18] included: patient age, gravidity, parity, gestational age, uterine size large for gestational age, vaginal bleeding, theca-lutein ovarian cyst, pre-evacuation hCG level, hemorrhage, hyperemesis, preeclampsia, hyperthyroidism, ≥2 complications, and acute respiratory distress syndrome (ARDS).

Univariate associations between patient characteristics and initial treatment type were evaluated using the chi-square or Fisher’s exact test for categorical variables and the Mann–Whitney test for continuous variables. To directly estimate risk ratios for binary outcomes, multivariate analyses assessing the association of hysterectomy with GTN development and the need for chemotherapy were performed using Poisson regression with robust variance. Variables included in multivariate models were selected based on clinical relevance and prior literature, rather than automatic statistical procedures. For specific outcomes, ≥2 complications and pre-evacuation hCG were retained as confounders.

Associations of initial treatment with FIGO stage and risk score of GTN, as well as chemotherapy response, were analyzed using the Mann–Whitney and Fisher’s exact tests. Statistical significance was set at *p* < 0.05. Analyses were conducted using SPSS (version 21.0, SPSS Inc., Chicago, IL, USA) and R (version 3.4.4/2018, The R Foundation).

In accordance with the journal’s guidelines, we will provide our data for independent analysis by a team selected by the Editorial Board for additional verification or reproducibility of this study in other centers, if requested.

## 3. Results

**Figure 1:**
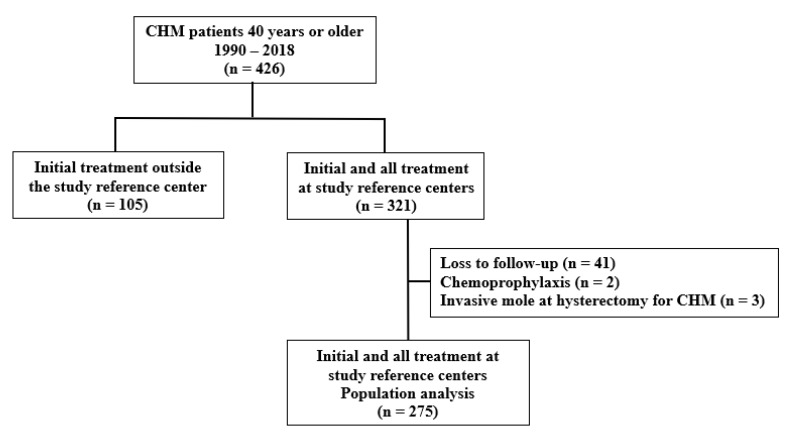
Flowchart of the study population showing women aged 40 years or older with complete hydatidiform mole.

Of 321 women with CHM aged ≥ 40 years registered during the study period, 2 received prophylactic chemotherapy at the time of uterine evacuation, 41 were lost to postmolar follow-up, and three were diagnosed with IM during hysterectomy. Thus, 275 were included in the analysis (Figure 1).

Hysterectomy was performed in 11% (31/275) of the patients. Table 1 shows the clinical characteristics of all study participants. Median patient age was 44 years. Median gestational age at CHM diagnosis was 11 weeks. Two or more clinical complications at presentation were observed in 12% (33/275) of participants. CHM presenting with associated gynecological disorders (uterine fibroids, adenomyosis, cervical intraepithelial neoplasia) were observed in 54.8% of patients who underwent hysterectomy. Median pre-evacuation hCG was 235,497 IU/L. GTN was observed in 27.6% (76/275).

As presented in Supplementary Table S1, 7.9% of patients with post-molar GTN were found to have metastatic disease, with no statistically significant difference observed between those initially managed with uterine evacuation and those managed with hysterectomy.

Univariate analysis revealed that, compared to women who underwent uterine evacuation (n = 244), those treated with hysterectomy for CHM (n = 31) were older (47 × 44 years, *p* = 0.001), had a higher number of pregnancies (5 × 3, *p* = 0.001), higher median pre-evacuation hCG levels (373,790 vs. 215,276, *p* = 0.007), and more often presented with enlarged uterus for gestational age (60% vs. 35%, *p* = 0.028), and with ≥2 medical complications at presentation (22.6% vs. 10.7%, *p* = 0.054). Of the 31 women who underwent hysterectomy, 3 met FIGO diagnostic criteria for GTN. The incidence of GTN was significantly higher after uterine evacuation than after hysterectomy (29.9% vs. 9.6%, *p* = 0.018). Likewise, the need for chemotherapy was markedly lower among patients treated with hysterectomy compared with those who underwent uterine evacuation (6.4% vs. 28.6%, *p* = 0.007).

Notably, 29% (9/31) of women initially treated with hysterectomy had previously undergone uterine evacuation to reduce vascular congestion and uterine size (median time between procedures of 8 days, minimum 5; maximum 10), and that all of them presented with uterine size > 13 cm (median uterine size of 16 cm, min 14; max 20). None of these patients developed GTN.

Hysterectomy (n = 31) was associated with an 83% lower risk of GTN compared with uterine evacuation (n = 244) [RR = 0.17; 95% CI, 0.04–0.71; *p* = 0.015]. The presence of two or more medical complications at presentation was not significantly associated with GTN development [RR = 0.94; 95% CI, 0.49–1.78; *p* = 0.849]. By contrast, pre-evacuation hCG levels were strongly associated with GTN risk: patients with hCG > 1,000,000 IU/L had an 18-fold higher risk compared with those with hCG < 100,000 IU/L [RR = 18.42; 95% CI, 5.24–64.78; *p* < 0.001] (Table 2).

Table 3 shows the multivariate analysis of factors associated with the need for chemotherapy in all patients (31 treated with hysterectomy and 244 with uterine evacuation). Hysterectomy was associated with a 92% lower risk of requiring chemotherapy compared with uterine evacuation [RR: 0.08 (0.01–0.64), *p* = 0.016]. In contrast, the risk of requiring chemotherapy increased significantly with higher pre-evacuation hCG levels. Patients with hCG > 1,000,000 IU/L had an 18.4-fold greater risk than those with hCG < 100,000 IU/L [RR: 18.4 (5.2–64.9), *p* = 0.001]. The presence of ≥2 medical complications at presentation was not significantly associated with chemotherapy requirement.

Of the 76 GTN patients, 4 underwent hysterectomy and required no chemotherapy to achieve sustained remission. Of the remaining 72 patients, who received chemotherapy, 23.6% (17/72) showed resistance to first-line treatment, 4.1% (3/72) relapsed after treatment with a single agent or sequential single-agent therapy, and 18.1% (13/72) required multiple agents for sustained remission. Median time to hCG normalization, starting from first-line chemotherapy initiation or hysterectomy for GTN, was 56 days (39.0–98.0). There was no significant difference in FIGO staging (*p* = 0.221) or risk score (*p* = 0.576) between groups. Resistance to first-line chemotherapy (17/72; 23.6%) and relapse (3/72; 4.1%) were observed only with uterine evacuation (Supplementary Table S1).

Of the 31 hysterectomies performed, 45.1% (14) led to some surgical complications. Need for packed red blood cell transfusion (Grade II) was the most frequent (5/14; 35.7%). Notably, intraoperative bladder injury (Grade IIIa) occurred in two patients, who required long-term urinary catheterization (15; 21 days). Total hysterectomy was the preferred type of surgery (29/31; 93.5%), while laparotomy was the most frequent route used (28/31; 90.3%) (Table 4).

## 4. Discussion

This study evaluated the impact of initial management with hysterectomy versus uterine evacuation on the development of postmolar GTN and the subsequent need for chemotherapy in women aged 40 years or older with complete hydatidiform mole. Our findings demonstrate that hysterectomy was associated with a significantly lower risk of GTN and reduced need for chemotherapy when compared with uterine evacuation. These results highlight the potential role of hysterectomy as an alternative initial treatment option in this patient population, particularly for those who no longer wish to preserve fertility. While oncologic outcomes were comparable between the two approaches, the surgical dimension must also be considered, as hysterectomy was associated with procedure-related complications, most commonly the need for packed red blood cell transfusion.

Similarly to previous reports [7,10,11], univariate analysis showed that the number of pregnancies, gestational age at CHM diagnosis, rate of uterine size exceeding gestational age, pre-evacuation hCG > 100,000 IU/L, and clinical complications at presentation were significantly higher in the hysterectomy group. On multivariate analysis, both the risk of GTN and the risk of requiring chemotherapy were significantly lower with hysterectomy compared to uterine evacuation.

In our cohort, the risk of GTN was 83% lower with hysterectomy than with uterine evacuation, consistent with earlier reports [7,10]. By contrast, Giorgione et al. (2017) [11] reported GTN rates of 58.3% after hysterectomy and 29.7% after uterine evacuation, suggesting no protective effect of hysterectomy in this age group. However, their cohort included patients with IM and PHM, resulting in a more heterogeneous population that may explain the higher GTN rates reported after hysterectomy. More recently, Zhao et al. (2019) conducted a systematic review and meta-analysis including older women with HM, which demonstrated that total hysterectomy significantly reduced the risk of post-molar GTN compared with uterine evacuation (OR 0.19; 95% CI, 0.08–0.48), with low heterogeneity across studies [19]. Taken together, these findings highlight the limitations of earlier single-center series with small or heterogeneous cohorts and support the greater external validity of our multicenter study, which is the most recent to focus exclusively on women aged ≥ 40 years with CHM. Moreover, our results are aligned with the updated international guidelines (EOTTD–ESGO–ISSTD, FIGO 2025) [8,9], which emphasize individualized treatment decisions in this high-risk group.

The need for chemotherapy was significantly lower among patients undergoing hysterectomy, with only 6.4% requiring treatment—representing a 92% lower than that observed after uterine evacuation. Moreover, 7.9% of our CHM patients had metastatic disease, consistent with previously reported rates of 10–12% in women of all ages [20,21]. These findings corroborate that most GTN cases are nonmetastatic and support the rationale that hysterectomy may either reduce or eliminate tumor bulk, thereby preventing GTN. In contrast, Savage et al. (2013) [3] reported that 13.6% of women with CHM required chemotherapy, with risk exceeding 20% in those aged 40 years or older.

The mechanisms underlying the increased risk of GTN and need for chemotherapy in older women remain unclear. While maternal age is a well-recognized risk factor, Savage et al. (2013) [3] suggested that changes in sperm characteristics with paternal aging may influence malignant potential, as CHM results from androgenetic conceptions. Additionally, heterozygous moles—resulting from an empty egg fertilization by two sperm—may carry a higher risk of malignant transformation, and their proportion may increase with maternal age [21,22].

GTN outcomes did not significantly differ between study groups. Resistance to first-line chemotherapy, relapse rate, and time to remission were comparable, consistent with data regarding the overall low-risk GTN population [21,22]. However, the small size of our hysterectomy group limits definitive conclusions.

Surgical complications of hysterectomy for CHM occurred in over 45% of cases, with the most common Clavien–Dindo complication being the need for packed red blood cell transfusion. Notably, 29% of these women had previously undergone uterine evacuation, with a median interval of eight days between procedures, and all had a significantly enlarged uterus. This underscores the prominent uterine vasculature in molar pregnancy and highlights the rationale for performing evacuation during or shortly before hysterectomy. By reducing both vascular congestion and uterine size, evacuation can facilitate a safer hysterectomy [23].

In low-risk nonmetastatic GTN patients treated with hysterectomy, Bolze et al. (2018) [24] observed no grade III, IV, and V surgical complications. In our study, complications of grades IV and V were also absent. However, grade III complications occurred in 14.3% of cases, which is high compared to the 1–3% rate reported after hysterectomy for benign diseases [25] and likely reflects the small number of women in our hysterectomy group.

Our CHM patients in the hysterectomy group were significantly older, and over half of them presented with associated gynecological disorders. These factors likely influenced the treatment decision for hysterectomy and highlight the importance of careful patient selection to ensure that hysterectomy is reserved for those most likely to benefit, while considering potential risks and long-term consequences.

Some limitations should be acknowledged in this study. Its retrospective design may have introduced biases affecting the interpretation of findings. The imbalance between groups, although reflective of real-world distribution, may have impacted the precision of estimates and reduced the statistical power of comparisons. The sample size also limited the ability to account for all potential confounders when estimating differences in GTN risk and chemotherapy need between groups. While the most clinically relevant confounders were included, a more comprehensive adjustment could have improved specificity in assessing the effect of initial treatment. However, adjusting for multiple confounders could also reduce the precision of estimates. In addition, although cases of invasive mole diagnosed at hysterectomy were excluded, it was not possible to completely rule out the presence of invasive mole in patients treated with uterine evacuation, and this possibility should be considered when interpreting our results.

Despite these limitations, this study has notable strengths. Excluding uterine evacuations performed outside study centers helped minimize potential bias, as such cases often present with abnormal hCG curves, which could lead to an overestimation of GTN frequency. Additionally, the multicenter design enhances the generalizability of findings, offering a broader perspective on CHM management in older women.

Overall, our results suggest that hysterectomy reduces GTN risk and the need for chemotherapy in older women with CHM, supporting its role as an alternative option when fertility preservation is not desired. However, despite helping prevent post-molar GTN by removing or decreasing local uterine invasion [19], hysterectomy does not guarantee a cure and has its drawbacks. Alongside the risk of surgical complications, individual patient risk factors—such as overlapping conditions including hypertension, obesity, and dyslipidemia—should also be evaluated during the decision-making process. Furthermore, in women < 45 years, even with ovarian preservation, hysterectomy can lead to early menopause and its related health consequences, including elevated cardiovascular risk, particularly stroke [26,27].

## 5. Conclusions

CHM initial treatment with hysterectomy was associated with lower GTN frequency and reduced need for chemotherapy in women ≥ 40 years. However, shared decision-making about surgery should be tailored to each patient and their risk factors and preferences. Future prospective studies with larger cohorts are warranted to better define which patients would derive the greatest benefit from this approach and to further clarify its long-term outcomes in CHM management.

## Figures and Tables

**Table 1:** Clinical characteristics of patients with complete hydatidiform mole ≥ 40 years according to initial treatment type (hysterectomy versus uterine evacuation).

		Initial Treatment		
Variable	Overall (n = 275)	Hysterectomy (n = 31)	Evacuation (n = 244)	*p*
Age (years)	44.0 (42.0–47.0)	47.0 (45.0–51.0)	44.0 (41.0–46.0)	0.001 ^(1)^
Gravidity	3.0 (3.0–5.0)	5.0 (3.0–6.3)	3.0 (3.0–5.0)	0.001 ^(1)^
Parity	2.0 (1.0–3.0)	2.0 (1.0–4.0)	2.0 (1.0–2.0)	0.007 ^(1)^
Presenting gynecological disorders	NA	17/31 (54.8%)	NA	-
Uterus fibroids		12 (38.7%)		
Adenomyosis		4 (12.9%)		
Cervical intraepithelial neoplasia		1 (3.2%)		
Gestational age (weeks)	11.0 (10.0–13.0)	13.0 (10.0–15.0)	11.0 (10.0–13.0)	0.064 ^(1)^
Uterine size > GA ^(a)^	94 (37.2%)	12 (60.0%)	82 (35.2%)	0.028 ^(2)^
Vaginal bleeding ^(b)^	203 (75.7%)	20 (69.0%)	183 (76.6%)	0.367 ^(2)^
Theca lutein cysts	22 (8.0%)	5 (16.1%)	17 (9.8%)	1.000 ^(2)^
Pre-evacuation hCG (IU/L)	235,497	373,790	215,276	0.007 ^(1)^
	(93,228–494,355)	(243,440–976,112)	(84,469–475,271)	
Hemorrhage	27 (9.8%)	3 (9.7%)	24 (9.8%)	1.000 ^(3)^
Hyperemesis	84 (30.5%)	5 (16.1%)	79 (32.4%)	0.096 ^(2)^
Preeclampsia	20 (7.3%)	5 (16.1%)	15 (6.1%)	0.059 ^(2)^
Hyperthyroidism ^(c)^	25 (9.4%)	8 (27.6%)	17 (7.2%)	0.001 ^(2)^
ARDS	2 (0.7%)	0 (0.0%)	2 (0.8%)	1.000 ^(3)^
≥2 medical complications at presentation	33 (12.0%)	7 (22.6%)	26 (10.7%)	0.054 ^(2)^
GTN	76 (27.6%)	3 (9.6%)	73 (29.9%)	0.018 ^(2)^
Required chemotherapy for remission	72 (26.1%)	2 (6.4%)	70 (28.6%)	0.007 ^(3)^
Time to remission (*)	69.0 (51.5–90.0)	80.0 (70.5–107.5)	65.0 (49.0–89.0)	0.002 ^(1)^

Data reported as median (IQR) or n (%). (*) Patients with spontaneous remission: interval between first uterine evacuation or hysterectomy and hCG normalization; ^(1)^ Mann–Whitney; ^(2)^ chi-square; ^(3)^ Fisher’s exact test: ^(a)^ Date of last menstrual period unknown by 11 patients treated with hysterectomy and 11 patients treated with uterine evacuation. ^(b)^ Data is missing for 2 patients treated with hysterectomy and 5 patients treated with uterine evacuation. ^(c)^ Data is missing for 2 patients treated with hysterectomy and 8 patients treated with uterine evacuation. GA: gestational age; ARDS: acute respiratory distress syndrome; GTN: gestational trophoblastic neoplasia. NA: Not available.

**Table 2:** Multivariate Poisson regression analysis of factors associated with GTN development among patients with complete hydatidiform mole and age ≥ 40 years.

Variable	RR	95% CI		*p*
Hysterectomy	0.17	0.04	0.71	0.015
≥2 medical complications	0.94	0.49	1.78	0.849
>1,000,000 IU/L	18.42	5.24	64.78	<0.001
(750,000–100,000] IU/L	9.48	2.11	42.70	0.003
(50,000–750,000] IU/L	9.67	2.65	35.25	0.001
(250,000–500,000] IU/L	8.06	2.37	27.34	0.001
[100,000–250,000] IU/L	5.48	1.59	18.82	0.007
hCG preevacuation (Ref: <100,000 IU/L)	1			

**Table 3:** Multivariate Poisson regression analysis of factors associated with the need for chemotherapy among patients with complete hydatidiform mole.

	RR	95% CI		*p*
≥2 medical complications	0.97	0.51	1.87	0.938
>1,000,000 IU/L	18.43	5.23	64.93	0.001
(750,000–100,000] IU/L	9.47	2.10	42.62	0.003
(50,000–750,000] IU/L	8.71	2.35	32.27	0.001
(250,000–500,000] IU/L	7.73	2.27	26.32	0.001
[100,000–250,000] IU/L	4.81	1.38	16.73	0.014
hCG pre-evacuation (Ref: <100,000 IU/L)	1			
Hysterectomy	0.089	0.012	0.640	0.016

**Table 4:** Hysterectomy surgical complications (Clavien–Dindo class), type and route among patients with complete hydatidiform mole and age ≥ 40 years initially treated with hysterectomy (n = 31).

	N	%
**Occurrence of complications**	14	45.1
**Type of complication**		
Blood transfusion–Grade II	5	35.7
Bladder injury/intraoperative repair–Grade IIIa	2	14.3
Postoperative paralytic ileus–Grade I	1	7.14
Changed procedure *–Grade I	1	7.14
Pulmonary embolism–Grade II	1	7.14
Atelectasis/lung physiotherapy–Grade I	1	7.14
Incisional hematoma–Grade I	1	7.14
Vaginal vault granulation tissue ^#^–Grade I	1	7.14
Urinary infection–Grade II	1	7.14
**Type of hysterectomy**		
Total	29	93.5
Subtotal	2	6.5
**Route of hysterectomy**		
Laparotomy	28	90.3
Laparoscopy	3	9.7

* Changed procedure–laparoscopic hysterectomy converted to laparotomy hysterectomy; ^#^ Required silver nitrate cauterization.

## Data Availability

The original contributions presented in this study are included in the article/Supplementary Materials. Further inquiries can be directed to the corresponding author.

## Supplementary Materials

The following supporting information can be downloaded at https://www.mdpi.com/article/10.3390/cancers17193125/s1, Table S1: Oncologic and therapeutic profile of patients with postmolar gestational trophoblastic disease ≥ 40 years, stratified by initial treatment approach (primary hysterectomy versus uterine evacuation).
