# Supplementary material for: Initial Treatment and Outcomes of Complete Hydatidiform Mole in Women 40 Years or Older: A Multicenter Cohort Study
Source: Cancers (Basel). 2025 Sep 26;17(19):3125. doi: 10.3390/cancers17193125 (PMC12524322; doi:10.3390/cancers17193125)
Supplement: Supplementary file 1 [file cancers-17-03125-s001.zip › cancers-3885785-supplementary.pdf]

Supplementary Table S1. Oncologic and therapeutic profile of patients with postmolar gestational trophoblastic disease ≥ 40 years, stratified by initial treatment approach (primary hysterectomy versus uterine evacuation).

Variable	Overall (n=76)	Hysterectomy (n=3)	Evacuation (n=73)	*p*
Stage				
I	70(92.1%)	2(66.6%)	68(93.2%)	*0.221* (2)
II	1(1.4%)	0(0.0%)	1(1.4%)	
III	5(6.5%)	1(33.4%)	4(5.5%)	
FIGO# Risk score	3.0(2.0-5.0)	3.0(2.0-x)	3.0 (2.0-5.0)	*0.576* (1)
Low-risk (FIGO 0-6)	61(80.2%)	2(66.6%)	59(80.8%)	*0.488* (2)
High-risk (FIGO 7-10)	15(19.8%)	1(33.4%)	14(19.2%)	
GTN treatment				
Required chemotherapy for remission				
No	4 (5.2%)	1 (33.3%)	3 (4.1%)	*0.151* (2)
Yes	72 (94.8%)	2 (66.4%)	70 (95.9%)	
Type of chemotherapy				*0.330* (2)
Single agent	59(81.9%)	1 (50.0%)	58 (82.9%)	
Multiple agent *	13(18.1%)	1 (50.0%)	12 (17.1%)	
Response to chemotherapy				
Resistance to first line chemotherapy	17(23.6%)	0(0.0%)	17(24.3%)	*1.000* (2)
Relapse	3(4.1%)	0(0.0%)	3(4.3%)	*1.000* (2)
Time to remission **	56.0(36.0-97.0)	92.0	56.0(37.0-99.0)	*0.694* (1)

(1) Mann-Whitney test
(2) Fisher's exact test

Data are expressed as median (IQR) or n (%).

FIGO. International Federation of Gynecology and Obstetrics

* Required multi-agent chemotherapy in all lines of treatment (1st, 2nd or 3rd line).

**Interval between first-line chemotherapy initiation or hysterectomy and hCG normalization.
